# Current Therapies in Clinical Trials of Parkinson’s Disease: A 2021 Update

**DOI:** 10.3390/ph14080717

**Published:** 2021-07-25

**Authors:** E. Maruthi Prasad, Shih-Ya Hung

**Affiliations:** 1 Graduate Institute of Acupuncture Science, China Medical University, Taichung 40402, Taiwan; emaruthip@gmail.com; 2 Department of Medical Research, China Medical University Hospital, Taichung 40447, Taiwan

**Keywords:** α-synuclein, clinical trials, dopamine receptor agonists, gene therapy, levodopa, Parkinson’s disease, plasma therapy

## Abstract

Parkinson’s disease (PD) is a progressive neurodegenerative disorder that currently has no cure, but treatments are available to improve PD symptoms and maintain quality of life. In 2020, about 10 million people worldwide were living with PD. In 1970, the United States Food and Drug Administration approved the drug levodopa as a dopamine replacement to manage PD motor symptoms; levodopa-carbidopa combination became commercialized in 1975. After over 50 years of use, levodopa is still the gold standard for PD treatment. Unfortunately, levodopa therapy-induced dyskinesia and OFF symptoms remain unresolved. Therefore, we urgently need to analyze each current clinical trial’s status and therapeutic strategy to discover new therapeutic approaches for PD treatment. We surveyed 293 registered clinical trials on ClinicalTrials.gov from 2008 to 16 June 2021. After excluded levodopa/carbidopa derivative add-on therapies, we identified 47 trials as PD treatment drugs or therapies. Among them, 19 trials are in phase I (41%), 25 trials are in phase II (53%), and 3 trials are in phase III (6%). The three phase-III trials use embryonic dopamine cell implant, 5-HT_1A_ receptor agonist (sarizotan), and adenosine A_2A_ receptor antagonist (caffeine). The therapeutic strategy of each trial shows 29, 5, 1, 5, 5, and 2 trials use small molecules, monoclonal antibodies, plasma therapy, cell therapy, gene therapy, and herbal extract, respectively. Additionally, we discuss the most potent drug or therapy among these trials. By systematically updating the current trial status and analyzing the therapeutic strategies, we hope this review can provide new ideas and insights for PD therapy development.

## 1. Introduction

Parkinson’s disease (PD) is a chronic progressive movement disorder that is the second leading cause of neurodegenerative diseases after Alzheimer’s disease. PD diagnosis is mainly based on brain imaging, neurological signs, and clinical symptoms [1]. Dopaminergic neuronal death in the substantia nigra of the midbrain causes striatal dopamine deficiency, which response to PD motor symptoms [2]. The motor symptoms in PD patients include slowness of movement, rigidity, tremor, freezing, muscle cramps, and dystonia [3]. The onset age of idiopathic (typical) PD patients is between 65 and 70 years old [4]. About 5% to 10% of patients acquire early-onset PD, which onsets before 50 years old [5]. Early-onset PD is often inherited and related to specific genetic mutations [6]. In PD-related genes, α-synuclein is a presynaptic neuronal protein (14–19 kDa) that regulates synaptic integrity and cellular functions [7]. α-Synuclein is one of the pathogenic hallmarks in PD; α-synuclein accumulates in Lewy bodies and associates with neuroinflammation during PD progression [8].

Using levodopa to improve PD motor symptoms is a gold standard. Levodopa was synthesized by a polish biochemist Casimir Funk in 1911 [9]. In 1961, levodopa was clinically examined in 20 PD patients by Walter Birkmayer, and he observed miraculous motor improvement for a few hours. In 1968, George Cotzias et al. used the oral form of levodopa tested in 28 PD subjects and revealed successful data for motor improvements [9]. In 1970, the United States Food and Drug Administration approved levodopa as a dopamine replacement to manage PD motor symptoms [9]. Subsequently, the first combined form of levodopa and carbidopa was used for controlling the motor symptoms in 1975 [10]. In combined form, levodopa converts dopamine by dopamine decarboxylase, activating dopamine receptors responsible for improving motor functions in the central nervous system and peripheral circulation [11]. Carbidopa acts as a decarboxylase inhibitor to facilitate levodopa availability in the brain [11]. The most common side effects of levodopa plus carbidopa combination include nausea, motor problems, hallucinations, depression, low blood pressure, irregular sleep, and gambling compulsions [12,13,14]. For more than 50 years, levodopa is still a standard in PD drug treatment. Unfortunately, levodopa treatment-induced dyskinesia and OFF symptoms remain unresolved; one report shows that dyskinesia and OFF symptoms occur in 13.5% and 55.9% of the study population, respectively [15]. The mechanism of long-term levodopa therapy-induced dyskinesia is not fully clear [16]. It indicates the urgent need for exploring new therapeutic strategies for PD treatment.

**Figure 1:**
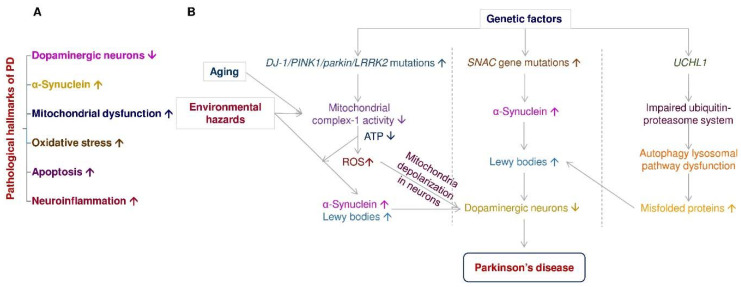
The pathological hallmarks of PD and the interplay of aging, environmental hazards, and genetics in the pathogenesis of PD. (**A**) The pathological hallmarks of Parkinson’s disease include dopaminergic neuronal death, α-synuclein aggregates, mitochondrial dysfunction, reactive oxygen species, apoptosis, and neuroinflammation. (**B**) PD is a multifactorial disorder involving aging, genetics, and environmental factors, associated with dopaminergic neuronal death. Gene mutations associate with PD includes *DJ-1*, *PTEN-induced putative kinase-1* (*PINK1*), *parkin*, *leucine-rich repeat serine/threonine kinase-2* (*LRRK2*), *Synuclein alpha* (*SNAC*), and *ubiquitin carboxyl-terminal hydrolase-L1* (*UCHL1*). Neurotoxins (e.g., rotenone, paraquat) and gene mutations of *DJ-1*, *PINK1*, *parkin*, *LRRK2*, and *SNAC* induce mitochondria complex I inhibition, ATP depletion, reactive oxygen species (ROS) accumulation, mitochondria depolarization, and mitochondrial dysfunction in dopaminergic neurons to promote neuronal death in the substantia nigra of PD patients. Overexpression of *SNAC* gene mutations enhance α-synuclein aggregation in Lewy bodies of PD brain. *UCHL1* gene mutations impair ubiquitin-proteasome systems and further induce autophagy lysosomal pathway dysfunction, contributing to Lewy body formation in dopaminergic neurons.

Disease-modification and neuroprotection are important therapeutic strategies to improve PD motor symptoms via inhibition or slow-down dopaminergic neuronal death in the brain [17]. Currently, available PD medications are only short-lived, such as levodopa [18]. Many drugs or therapy have shown definite neuroprotection in dopaminergic neurons *in vivo* and *in vitro*; several failed when tested in clinical trials. Figure 1A depicts that dopaminergic neuronal death, α-synuclein aggregates, mitochondrial dysfunction, reactive oxygen species, apoptosis, and neuroinflammation are the pathological hallmarks of PD [19]. PD is a multifactorial disorder involving aging, genetics, and environmental factors. Figure 1B elucidates the impact of aging, environmental hazards, and genetic factors in PD progression.

**Figure 2:**
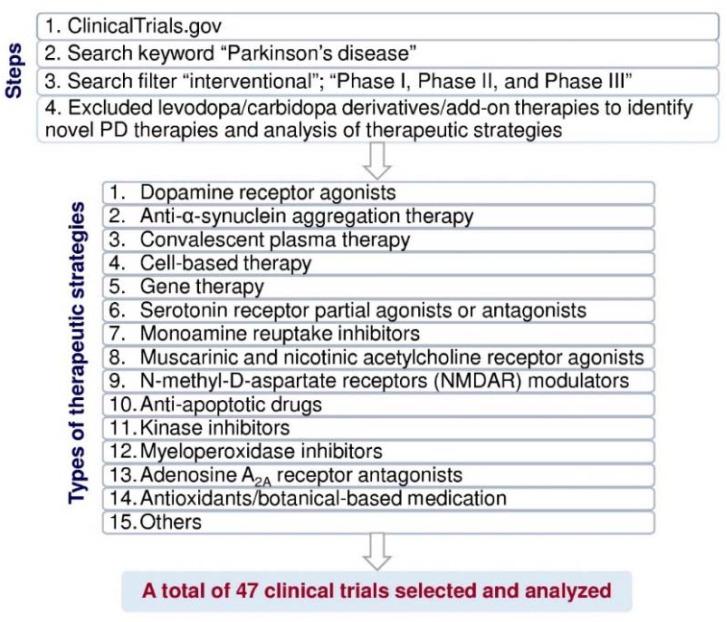
Flow diagram of the method used to select and analyze the clinical trial data for PD treatment from ClinicalTrials.gov (https://clinicaltrials.gov, accessed on 16 June 2021).

Evaluation of new drugs is monitored carefully in clinical trials. According to the United States Food and Drug Administration, the purposes of phase I are safety and dosage; approximately 70% of drug/therapy moves to phase II [20]. The purpose of phase II is to test efficacy and side effects; more or less 33% of drugs move to phase III [20]. Phase III is to study the potency and monitoring of adverse reactions [20]. In 2000, the United States National Library of Medicine created the web-based registry “ClinicalTrials.gov” for users to search the clinical trial information, including study design, methods, results, expected end dates, etc. The data is maintained or updated by sponsors worldwide. To date, the clinical trials’ registry consists of over 2700 PD clinical studies. Clinical trial outcomes/endpoints are considered comparative effectiveness research [21], and outcomes can be achieved using various strategies such as cognitive or behavioral scores, magnetic resonance imaging, positron emission tomography, electrophysiological monitoring, or biological biomarkers. Each clinical trial is developed and assessed for treatment benefits to prevent adverse events [21]. In clinical trials, post-approval is required for comparative research to compare clinical trials with available standard medicines/therapy, which provides quality of life, safety, and tolerance to obtain efficient data in the larger patients’ population [21]. Primary endpoints are essential and sufficient to establish the efficacy of a drug/therapy in clinical trials. Based on the primary endpoints, secondary endpoints are sufficient to claim/labeling the efficacy of the clinical trial study, and the exploratory/tertiary endpoints support descriptive information [22]. Levodopa is used to treat PD for over 50 years, and levodopa therapy-induced dyskinesia and OFF symptoms remain unresolved. Therefore, we urgently need to analyze each current clinical trial’s status and therapeutic strategy and discover new therapeutic approaches for PD treatment. In this review, we have screened the clinical trial pipeline data from Clinicaltrials.gov to analyze PD therapies. First, we excluded levodopa/carbidopa derivatives add-on therapy to select 293 clinical trials. Among those, we identified forty-seven clinical trials that belong to new PD therapeutic strategies by the following conditions and filters in Figure 2.

## 2. PD Therapeutic Strategies in Clinical Trials

**Figure 3:**
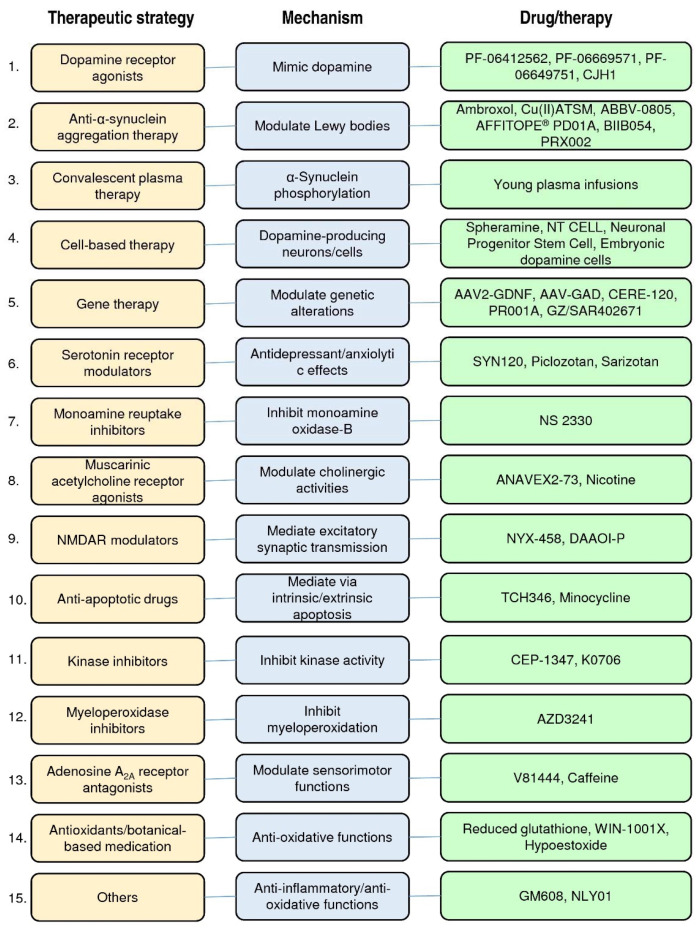
The type of therapeutic strategies, mechanism, and the current drugs/therapies in the clinical trials of PD treatment.

In Figure 2, we have classified these therapeutic strategies into 15 types: dopamine receptor agonists, anti-α-synuclein aggregation therapy, convalescent plasma therapy, cell-based therapy, gene therapy, serotonin receptor partial agonists or antagonists, monoamine reuptake inhibitors, muscarinic and nicotinic acetylcholine receptor agonists, N-methyl-d-aspartate receptor (NMDAR) modulators, anti-apoptotic drugs, kinase inhibitors, myeloperoxidase inhibitors, adenosine A_2A_ receptor antagonists, antioxidants/botanical-based medication, and others. Figure 3 depicts the type of drug or therapy, mechanisms, and the current drugs/treatments in PD.

**Figure 4:**
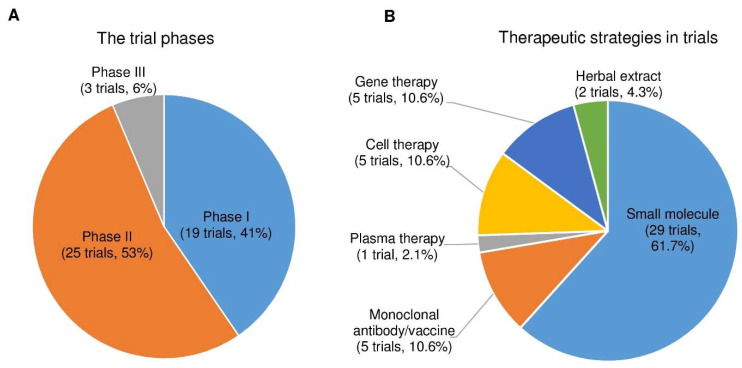
The trial phases and therapeutic strategies in the clinical trials for PD treatment. (**A**) A pie chart shows the individual percentage of phase I, phase II, and phase III trials to the total. The phase I/II or II/III trials on ClinicalTrials.gov are showed as phase I or II, respectively. (**B**) A pie chart shows the proportions of each therapeutic strategy to the total PD clinical trials.

After analyzing the data collected from ClinicalTrials.gov, we identified forty-seven registered interventional clinical trials in phases I, II, and III as new PD therapies based on the current trial status that shows ongoing/updated or discontinued as of 16 June 2021. Among the forty-seven trials, 19 trials (41%) in phase I, 25 (53%) in phase II, and 3 (6%) in phase III (Figure 4A; the phase I/II or II/III trials on ClinicalTrials.gov are considered phase I or II, respectively.)

Next, we divided the therapeutic strategy of each trial into the small molecule, monoclonal antibody, plasma therapy, cell therapy, gene therapy, and herbal extract. As a result, we found 29 clinical trials using small molecules (61.7%), 5 using monoclonal antibodies (10.6%), 1 using plasma therapy (2.1%), 5 using cell-based therapies (10.6%), 5 using gene therapies (10.6%), and 2 using herbal extracts (4.3%) (Figure 4B). 

**Figure 5:**
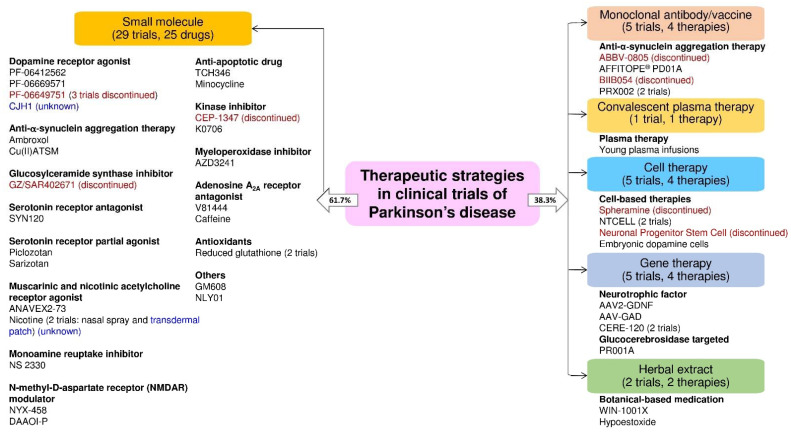
The drug or therapy with its therapeutic strategy and trial status in the clinical trials for PD treatment. The blue and red text color indicates the trial status is unknown and discontinued, respectively (active as of 16 June 2021).

The clinical trials data showed that the majority of the trials were small molecular therapeutic strategies in the PD trials. We found several PD therapies focused on neuroprotection and disease modification. Based on the trial status (ongoing, unknown, or discontinued), we found that the clinical trial status for the CJH1 (NCT01684475) and nicotine transdermal patch (NCT01560754) is unknown. Moreover, the clinical trials of PF06649751 (NCT02847650 and NCT02687542), GZ/SAR402671 (NCT02906020), CEP-1347 (NCT00040404), ABBV-0805 (NCT04127695), and BIIB054 (NCT03318523), spheramine (NCT00206687), and neuronal progenitor stem cell (NCT00927108) therapy were discontinued due to various reasons (Figure 5).

### 2.1. Dopamine Receptor Agonists

Dopamine receptor agonists are the main therapy class that mimics dopamine function in PD patients [23,24]. All the dopamine receptors are G protein-coupled receptors containing D_1_ and D_2_ types; they pair with Gs of G proteins, which trigger the adenylyl cyclase system and consequently rouse the cAMP synthesis [25]. D_1_ receptor contains D_1_ and D_5_ subtypes; D_2_ receptor contains D_2_, D_3_, and D_4_ subtypes [26,27]. Although upon the prolonged oral dopamine receptor agonists, treatment becomes unresponsive [16,28]. 

We found six clinical trials that used four small molecular drugs that act as a dopamine receptor agonist in PD treatment. PF-06412562 is a moderately potent, highly selective oral D_1_/D_5_ dopamine receptor partial agonist; PF-06412562 has good selectivity than other dopamine receptor subtypes [29]. In a phase I trial, oral administration PF-06412562 had potential antiparkinsonian efficacy in 13 PD patients without significant acute changes in cardiovascular parameters reported with previous D_1_ agonists (Table 1) (NCT03665454) [29]. The trial results revealed that the PF-06412562 was well tolerated in advanced PD patients and met the primary and secondary endpoints [30]. PF-06669571 is a novel and oral D_1_/D_5_ dopamine receptor partial agonist with a non-catechol-based structure that has demonstrated efficacy in preclinical models of PD symptoms [31]. Gurrell et al. (2018) showed that PF-06669571 (1 or 3 mg, four times a day) in the phase I trial was safe and well-tolerated with minor adverse events such as nausea in 10 idiopathic PD patients (Table 1) (NCT02565628) [31]. PF-06649751 is also a novel, oral, non-catechol, D_1_/D_5_ dopamine receptor partial agonist [32]. In phase I study, PF-06649751 showed safety, efficacy, and tolerability with pharmacological profile; further phases of trials on a larger scale were carryout by Pfizer (Table 1) (NCT02224664) [32]. In addition, the phase II study PF-06649751 (1 daily oral dose) demonstrated significant motor balance improvement and was well-tolerated in 25 early-stage PD patients (NCT02847650) [33]. The study showed PF-06649751 treatment had the most common adverse events, including nausea, headache, dry mouth, somnolence, and tremor [33]. The phase II trials of PF-06649751 failed to demonstrate efficacy in moderate/advanced PD and were terminated (Table 1) (NCT02847650 and NCT02687542). The D_2_-specific dopamine receptor agonist CJH1 (CLR4001) is undergoing a phase I/II clinical trial developed by Alexandra Marine and General Hospital, and the current trial status has not been updated since 2012 (Table 1) (NCT01684475). Moreover, the clinical data of PF-06412562 ((4-[4-(4,6-dimethyl-5-pyrimidinyl)-3-methylphenoxyl]-1H-pyrazolo [4,3-c]pyridine) showed safety and tolerability against advanced PD (NCT03665454) [30]. The PF-06669571 treatment was also reported as safe and tolerable against idiopathic PD [31]. Among the three trials of PF-06649751, two (NCT02847650 and NCT02687542) were discontinued due to insufficient efficacy, and one trial shows phase I, but no post updated since March 2017 (NCT02224664).

Long-term (1–5 years) usage of dopamine receptor agonists offers adverse effects based on the dosage. The side effects include nausea, vomiting, irregular heartbeats, low blood pressure, renal/pulmonary diseases, and dizziness [34]. Besides, extended dopamine receptor agonist usage causes chorea/dystonic movements, illusions, delusions, mania, mental disturbances, yawning, and irresistible sleep periods [34]. Several studies revealed that dopamine receptor agonists aid in drug-induced mental illness, anxiety, anorexia nervosa, schizophrenia, hypersexuality, gambling mentality, and compulsive shopping disorders [35]. Among currently used dopamine receptor agonists for PD treatment, pramipexole treatment might have maximum side effects [36].

### 2.2. Anti-α-Synuclein Aggregation Therapy

α-Synuclein, an unfolded highly soluble protein in presynaptic neurons of the brain [37]. α-Synuclein aggregation is a pathologic hallmark of synucleinopathies in sporadic and hereditary PD [38]. Aggregation of α-synuclein induces many pathological conditions such as autophagy or lysosomal disorder, synaptic dysfunction, mitochondrial dysfunction, endoplasmic reticulum stress, and oxidative stress [39,40]. All the pathological conditions further lead to proteinaceous cytoplasmic inclusions, known as Lewy bodies and Lewy neurites [40]. Anti-α-synuclein aggregation treatment mainly increases the cellular clearance mechanisms and regulates Lewy bodies [41].

In anti-α-synuclein aggregation therapies, we found five clinical trials that use monoclonal antibodies (ABVV-0805, BIIB054, and PRX002) or vaccines (AFFITOPE^®^ PD01A), and two trials use small molecules (ambroxol and Cu(II)ATSM) in the clinical trials for PD. In a phase I trial, monoclonal antibody ABVV-0805 has been withdrawn due to strategic considerations by AbbVie (Table 2) (NCT04127695). BIIB054 (cinpanemab) is an IgG_1_ protein produced from memory B cells from aged people without nervous system diseases [42]. BIIB054 treatment showed an 800-fold higher affinity towards binding to α-synuclein that inhibited spread or aggregation and improved motor balances [42]. The current status of the BIIB054 (phase II) study shows termination due to not meeting the primary and secondary endpoints for the treatment of PD (Table 2) (NCT03318523).

Anti-α-synuclein monoclonal antibody PRX002 (RO7046015/prasinezumab) is developing (phase II) by Hoffmann-La Roche (NCT03100149). In addition, a phase-I study used PRX002 that targets anti-α-synuclein is developing by Prothena Bio-sciences Limited (Table 2) (NCT02157714). Jankovic et al. (2018) showed that single and multiple doses of the anti-α-synuclein monoclonal antibody PRX002 were generally safe and well-tolerated, and it resulted in robust binding of peripheral α-synuclein and dose-dependent increases of PRX002 in cerebrospinal fluid (Table 2) (NCT03100149) [43]. AFFITOPE^®^ PD01A is a phase I experimental vaccine made by short synthetic peptides to produce antibodies against α-synuclein aggregation and improve immunity, developed by Affiris AG (Table 2) (NCT01568099) [44]. The same vaccine AFFITOPE^®^ PD01A trials were also registered with NCT01885494, NCT02216188, and NCT02618941 by Affiris AG (Table 2) [44]. AFFITOPE^®^ PD01A demonstrated safe and well-tolerated in patients; the trials also showed increased antibodies in the blood and cerebrospinal fluid extended by Affiris AG for further studies [44,45]. Ambroxol is a potential disease-modifying small molecule that targets α-synuclein aggregation (Table 1). Since 1979, ambroxol has been using as a drug for cough [46]. A report indicates ambroxol blocks autophagy and driving cargo towards the secretory pathway [47]. To date, the ambroxol administration has not been reported for severe side effects; more or less, it may cause gastric ulceration [46]. Mullin et al. (2020) showed that ambroxol therapy could cross the blood-brain barrier, dock with the enzyme β-glucocerebrosidase, and decrease cerebrospinal fluid α-synuclein level in PD patients without glucocerebrosidase genetic mutations [48]. The preclinical data of ambroxol reduced α-synuclein aggregation and lowered the disease progression [48,49]. The Lawson Health Research Institute is developing ambroxol as an anti-PD drug. The current status is under subject recruitment at phase II without result, and the study is estimated to be completed by December 2021 (NCT02914366). Next, Copper (II) diacetylbis (N(4)-methyl thio semi carbazonato) (Cu(II)ATSM) is a small molecule permeable to the blood-brain barrier, which is currently in a phase I trial for PD treatment developing by Collaborative Medicinal Development Pty Limited. Cu(II)ATSM treatment improves neuroprotection by cognitive and motor performances via modulating brain metal levels and dopamine metabolism in animal models [50]. The trial status of Cu(II)ATSM shows that subject recruiting is complete, and no published data are available (Table 1) (NCT03204929).

Recent literature shows that the use of small molecules against α-synuclein aggregation is safe and efficient by increasing autophagy/lysosomal flux than other PD therapies [40], for instance, ambroxol or Cu(II)ATSM small molecule α-synuclein target drugs being tested in the pipeline of clinical trials, and could be successful drugs in future. In PD, α-synuclein aggregates induce neuronal loss, one of the key factors associated with neuroinflammation [51]. Nevertheless, the long-term usage of α-synuclein targeted therapies may also cause several side effects such as loss of synaptic functions and disruption of endoplasmic reticulum-Golgi stress [52].

### 2.3. Convalescent Plasma Therapy

Plasma contains antibodies, protein complexes, salts, and organic compounds. Therefore, plasma therapy has emerged as one of the safe and well-tolerated PD treatments [58]. It is well-known that infusion of the young plasma reduces α-synuclein and Lewy bodies. We found a phase I clinical trial study developing by Stanford University using the transfusion of young plasma (1 unit, twice a week for four weeks) into 15 moderate-stage PD patients (Table 2) (NCT02968433). The trial results show that young fresh frozen plasma was safe, feasible, well-tolerated in PD patients, with no serious adverse events, and the most common adverse effects were mild skin reactions during infusions [58]. In addition, the data showed young plasma maintained improvements in phonemic fluency and the stigma subscore of the PDQ-39 and reduced peripheral TNF-α [58].

α-Synuclein aggregation is not the only cause for PD progression; multiple factors are associated with the disease; hence plasma therapy may show limited effects [59]. Nevertheless, plasma therapy also causes health risks such as allergic reactions, breathing difficulties, infection to human immunodeficiency virus (HIV), hepatitis B or hepatitis C virus, or even may cause infection with unknown viruses [58,60].

### 2.4. Cell-Based Therapy

Cell therapy involves the introduction of dopamine-producing cells into the brain via transplantation. Cell-based therapy is a sustainable option that can reduce neurological inflammation in PD [61]. Spheramine (BAY86-5280) is a human retinal cultured epithelial pigment that produces levodopa [62]. In a phase II trial, 35 PD patients were transplanted spheramine (325,000 cells/side) into the post-commissural putamen by micro-carrier in 36 PD patients who underwent sham surgery (Table 2) (NCT00206687). The result showed spheramine did not have significant anti-parkinsonism effects (change in mean motor scores did not differ significantly between groups) [62]. In this trial, 2 and 7 patients died in the sham surgery and spheramine transplantation group, respectively [62]. Porcine choroid plexus produces several neurotrophins and can be safely delivered to the striatum in an encapsulated formulation to protect them from immune attack [63]. NTCELL is an alginate-coated capsule containing clusters of neonatal porcine choroid plexus cells [64]. In 2013, Living Cell technologies hosted a phase I/II trial of NTCELL implantation (Table 2). The trial status shows it was completed on 4 June 2020, and no published data are available (NCT01734733). The phase II trial results showed intra-striatal NTCELL implantation was safe and well-tolerated without change in Unified PD Rating Scale (UPDRS) motor scores for 26 weeks post-intervention compared with baseline (Table 2) (NCT02683629) [63]. In cell-based therapies, embryonic dopamine cell implant surgery entered into phase III trials by the University of Colorado Denver (Table 2) (NCT00038116). The published data of the embryonic dopamine cell surgery revealed that it benefits in reversing severe PD symptoms in younger patients, but not in older patients [65,66,67,68,69,70,71,72,73]. Neuronal progenitor stem cells are collected from the adult human brain to treat PD, Alzheimer’s disease, and multiple sclerosis by Rajavithi Hospital (Thailand) (NCT00927108). Recent information on the neuronal progenitor stem cell therapy clinical trial shows withdrawn by Rajavithi Hospital for unspecific reasons (NCT00927108). In cell therapy, the spheramine, and neuronal progenitor stem cells were discontinued from phase II. NTCELL and embryonic dopamine cell implant methods showed safety and tolerability against PD and could be effective. However, cell therapies may also cause immunosuppression and genetic changes that lead to genetic overexpression or carcinoma; or the treatment may be less effective with a higher risk of non-motor expressions depending on the patient [74].

### 2.5. Gene Therapy

Gene therapy for PD treatment includes genetically engineered therapeutic genes that actively replace, knockout, or correct the faulty genes in PD patients [75]. Various serotypes of non-replicating genetically engineered viral vectors, such as an adeno-associated virus (AAV) or lentivirus, have been applied in gene therapy [76]. Gene therapy can prevent dopaminergic neuronal death in the brain [77]. In PD, gene therapies mainly aim to increase the stimulation of neurotrophic action in the brain to improve motor balance in patients [78]. Besides, regulation of glucocerebrosidase levels by gene therapy is one potential therapeutic approach in treating PD [79].

We observed six clinical trials (five gene therapies and one small molecular) that use gene therapy for PD treatment, such as AAV2-GDNF, AAV-GAD, CERE-120 (2 trials), PR001A, and GZ/SAR402671. Presently, AAV2-GDNF (adeno-associated virus serotype 2 borne glial cell line-derived neurotrophic factor) is in a non-randomized open-label safety trial developing for PD treatment by Brain Neurotherapy Bio, Inc. (Table 2). In this phase I trial, they transfer AAV2-GDNF into putamen; the trial status shows under subject recruitment, no published data available (NCT04167540). In a phase I trial, subthalamic nucleus gene transfer by the AAV-GAD (adeno-associated virus borne glutamic acid decarboxylase) indicated safe, well-tolerated, and significant improvements in motor UPDRS scores in 12 advanced PD patients (Table 2) (NCT00195143) [80]. Bilateral stereotactic administration of CERE-120 (AAV2-neurturin) into the substantia nigra was safe and well-tolerated, CERE-120 is developing at phase I/II by Sangamo Therapeutics (Table 2) (NCT00985517) [81]. Besides, a piece of news published about the study showed that the treatment failed to improve the patients’ difficulties compared to the sham group [82]. Another phase II study of CERE-120 showed no significant difference between the treated and control groups (Table 2) (NCT00400634) [83]. They also observed adverse effects upon treatment with CERE-120 in 13 patients out of 38 total patients [83]. Besides, three of the patients from the CERE-120 treatment developed tumors. Overall, the study data showed no significant improvements compared to the control group [83]. Gene therapy using PR001A targets glucocerebrosidase activity. Preclinical data of PR001A administration also showed diminished α-synuclein aggregation and Lewy body levels markedly [84]. The treatment comprises one-time administration of the *GBA1* gene (encodes glucocerebrosidase) into the cisterna magna developing by Prevail Therapeutics. The current status of the PR001A phase I/IIa trial shows under subject recruitment, no data available, and is estimated to complete by June 2027 (Table 2) (NCT04127578). GZ/SAR402671 is a small molecular glucocerebrosidase gene mutating therapy in early PD, developing by Genzyme (Table 1). A phase II clinical trial assesses the safety and tolerability of oral GZ/SAR402671 for four weeks compared to placebo controls (NCT02906020). In addition, preclinical data of oral administration of GZ/SAR402671 inhibited lipid accumulation and slowed the progression of α-synuclein, ubiquitin, and tau phosphorylation, and enhanced cognitive deficits [53]. GZ/SAR402671 administration was safe and well-tolerated in the phase I trial [53], but the trial has been terminated due to not meeting the primary and secondary endpoints updated on 04 June 2021 (NCT02906020). Overall, the gene therapies targeting the *GBA1* gene (PR001A) or neurotrophic factors (AAV-GAD) may be successful in the future.

In gene therapy, delivering the desired gene by a suitable vector into the specific brain area is a sensitive and complicated task. For instance, AAV-based vectors have a limited capacity of 4.7-kb, which prohibits the integration of multiple genes [85]. Several risk factors of gene therapy include impaired gait, dorsal root ganglia, ataxia, and increased transaminases [86,87]. In some cases, the overexpression of the transgene may cause severe toxicity to the targeted organs or tissues [86,87].

### 2.6. Serotonin Receptor Agonists or Antagonists

Motor activities, depression, cognitive and autonomic functions are regulated by the serotonergic neurotransmission system [88]. Therefore, drugs targeting serotonergic receptors modulate behavioral qualities and improved motor balances [89]. We found three clinical trials use serotonin receptor agonists (piclozotan and sarizotan) or antagonists (SYN120) for PD treatment. Piclozotan (SUN N4057) is a selective 5-HT_1A_ receptor agonist developing at phase II by Daiichi Sankyo, Inc. A preclinical study showed that serotonin 1A receptor agonists piclozotan ameliorate motor performances in 6-hydroxydopamine-induced models [90]. The trial status shows it completed subject recruitment while results show no serious adverse events compared to the placebo cohort. However, the piclozotan treatment produced few minor adverse events such as headache, nausea, dizziness, and hypertension (Table 1) (NCT00623363). Preclinical literature also showed that sarizotan benefits in reducing dyskinesia, respiratory problems [91]. Sarizotan possesses serotonin receptor partial agonist and dopamine D_2_ receptor agonist activities, developing at phase III by EMD Sereno. The status of this trial shows no results (Table 1) (NCT00105508). A study shows that 2 mg/day sarizotan administration had no improvements in dyskinesia compared to placebo subjects [54]. SYN120 is a dual 5-HT_6/5_-HT_2_ serotonin receptor antagonist. An update from the American Academy of Neurology’s 2019 annual meeting (on 15 May 2019) declared that the SYN120 (SYNAPSE) trial in phase II studied with 80 patients has failed to improve cognitive performance (Table 1) (NCT02258152) [92].

Moreover, not all the serotonin receptor agonists are active/control in mediating the PD. In addition, some of the 5-HT_2B_ receptor agonists reported offering adverse effects; for instance, fenfluramine, pergolide, and cabergoline were discontinued from the pharmaceutical industry due to cardiac fibrosis [93].

### 2.7. Monoamine Reuptake Inhibitors

Pathogenesis of PD is associated with oxidative stress and monoamine oxidase-B activities in the glia of the brain [94]. Monoamine reuptake inhibitors prevent the reuptake of dopamine, serotonin, norepinephrine, and periphrastically. Together these events further activate the cholinergic system that represents potential PD therapeutic targets [95]. We found a clinical trial that uses a monoamine reuptake inhibitor (NS 2330) for PD treatment. NS 2330 (tesofensine) is a triple monoamine reuptake inhibitor. It inhibits the reuptake of cholinergic molecules of dopamine, serotonin, and norepinephrine. These cellular events indirectly stimulate the cholinergic system [96]. Presently, NS 2330 is developing at phase II by Boehringer Ingelheim. For this trial, they recruited 261 subjects with PD < 5 years that were not receiving dopaminergic treatment and randomly assigned them to daily with NS 2330 at 0.25 mg, 0.5 mg, 1.0 mg, or placebo (Table 1) (NCT00148486) [97]. Hauser et al.’s (2007) clinical trial data showed that NS 2330 treatment did not affect the total UPDRS score compared to placebo subjects during the fourteen-week experimental period [97]. Besides, the NS 2330 data were not shown to alter parkinsonian signs or dyskinesia when infused with levodopa [98].

### 2.8. Muscarinic and Nicotinic Acetylcholine Receptor Agonists

Cholinergic receptors contain muscarinic receptors (sensitive to muscarine) and nicotinic receptors (sensitive to nicotinic); they function in somatic and autonomic signal transductions in the nervous system [99]. We observed three trials that use muscarinic or nicotinic acetylcholine receptor agonists (Table 1). ANAVEX2-73 is a small molecule that binds to muscarinic acetylcholine and sigma1 (s1) receptors in the low micromolar range developing by Anavex Life Sciences Corp [100]. In October 2019, they started a phase II open-label extension to evaluate the effects of ANAVEX2-73 in 120 PD subjects with dementia on the safety and efficacy of daily treatment (Table 1) (NCT04575259). Anavex Life Sciences Corp. estimates the study completion by 31 October 2021. Nicotine is used in two phase-II trials as a transdermal patch (in 2012) and a nasal spray (in 2019) for PD treatment. Unfortunately, the trial data for the nicotine transdermal patch (7 or 14 mg for 52 weeks) in PD patients is unavailable (Table 1) (NCT01560754). Another trial using nicotine nasal spray trial status shows complete under subject recruitment and no results posted (Table 1) (NCT03865121). Although the high doses of transdermal nicotine were also tolerated, they failed to show significant improvements in UPDRS scores [101].

Moreover, the cholinergic drug treatment disadvantages might be less efficient than dopamine receptor agonists or carbidopa-levodopa treatment or even may cause adverse effects on parasympathetic nerve-related organs in PD [102].

### 2.9. N-Methyl-d-Aspartate Receptor (NMDAR) Modulators

In addition to the dopaminergic neuronal loss in PD, dysregulation of NMDAR in the cortical-striatal-pallidal-thalmo-cortical network and changes in plasticity of the brain regions are also crucial for cognitive function [103]. NMDAR modulators enhance synaptic plasticity [103]. We observed two trials in phase II that use NMDA receptor modulators (NYX-458 and DAAOI-P) in the PD treatment (Table 1). NYX-458 is an NMDAR modulator that increases cognitive properties and synaptic plasticity [103,104]. NYX-458 phase II trial plans to recruit PD subjects with mild cognitive impairments; the trial status shows the active recruiting and is estimated to complete by 22 December 2022 (NCT04148391). DAAOI-P (Flavoenzyme) is a D-amino acid oxidase inhibitor that facilitates the NMDA receptor subunit-1 and catalyzes/degrades D-amino acids by oxidative deamination in PD dementia developing by China Medical University Hospital (Taiwan). The phase II trial status of DAAOI-P in PD subjects with dementia shows under recruitment, and no published data is available; the trial is estimated to complete by July 2022 (NCT04470037).

NMDAR modulators also showed adverse effects such as irregular heartbeats, nausea, vomiting, psychosis, catalepsy, constipation, analgesia, and amnesia [105,106].

### 2.10. Anti-Apoptotic Drugs

During PD progression, degeneration of neurons occurs due to apoptosis and necrosis [19]. We observed two clinical trials that use small molecule anti-apoptotic drugs, i.e., TCH346 and minocycline. TCH346 is also known as dibenz[b,f]oxepin-10-ylmethyl-prop-2-ynyl-amine hydrogen maleate salt. TCH346 is undergoing phase I/II clinical trial in 301 early-stage PD patients, and it is developing by Novartis; the trial status shows it is complete, and no published data are available (Table 1) (NCT00407212). Preclinical data showed that TCH346 protected from dopaminergic neuronal damage [107]. Minocycline is a neuroprotective synthetic tetracycline derivative that mainly targets anti-apoptotic pathways; it modulates microglial cells and reduces oxidative stress and neuroinflammation [108]. Minocycline treatment (200 mg/day) has completed the phase II clinical trial in 66 subjects with early untreated PD patients (Table 1) (NCT00063193). The published trial data showed that the mean changes in total UPDRS scores for minocycline were not significantly different in PD treatment [55].

### 2.11. Kinase Inhibitors

LRRK2 kinase activities and their effects in PD enhance the degeneration of disease progression; LRRK2 inhibition offers neuroprotection in PD [109]. We identified two clinical trials that use small molecule kinase inhibitors (CEP-1347 and K0706). CEP-1347 (KT7515) is a semisynthetic inhibitor of the mixed lineage kinase family; it promotes neuronal survival by inhibiting c-Jun amino-terminal kinases (JNKs) activation [110]. CEP-1347 was in phase II/III trial and was terminated by Cephalon due to insignificant trial results (Table 1) (NCT00040404) [56,111]. The published trial results showed that urate and its determinants caused the disease progression [56]. Moreover, CEP-1347 treatment fails to slower disease progression in early PD patients [111]. K0706 is a potent orally selective inhibitor of cABL protein tyrosine kinase; K0706 exhibits neuroprotective activity [112]. A phase II trial using K0706 was performed by Sun pharma (SPARC) (Table 1). The trial status of K0706 in early PD subjects shows under subject recruitment with no results posted and is estimated to be completed by March 2023 (NCT03655236).

Kinase inhibitors may also cause side effects such as fatigue, diarrhea, hypertension, abnormal wound healing or rashes, periorbital edema, and myelosuppression [113,114].

### 2.12. Myeloperoxidase Inhibitors

Mitochondrial dysfunction, oxidative stress, and the formation of excessive reactive oxygen species are involved in the progress of PD neurodegeneration [115]. Myeloperoxidase inhibition reduces the production of reactive oxygen species and further neuroinflammation in PD [116]. We found a clinical trial that uses AZD3241 as a myeloperoxidase inhibitor. It is an irreversible myeloperoxidase inhibitor and was found to be safe without serious adverse events; the common side effects are nausea, headache, nasopharyngitis, insomnia, and weakness [115]. AZD3241 undergoes a phase II clinical trial (300 or 600 mg, two times a day for 12 weeks) in 51 PD subjects (by AstraZeneca). The trial status shows complete, and no published data is available (Table 1) (NCT01603069). Jucaite et al. (2015) showed that administration of AZD3241 (at 600 mg twice a day for eight weeks) inhibited 11C-PBR28 binding to the translocator proteins (a hallmark of microglial activation) and inflammation in the PD patients and might be effective against myeloperoxidation in PD [115].

### 2.13. Adenosine A_2A_ Receptor Antagonists

Adenosine receptors are promising therapeutic targets for a variety of diseases, including PD [117]. We found two clinical trials that use adenosine A_2A_ receptor antagonists (V81444 and caffeine). The V81444 is a small molecular drug for PD treatment in phase I clinical trial, developing by Vernalis (R&D) Ltd., Kansas, United States (Table 1). The trial status shows completed in subject recruiting, and the result is unavailable (NCT02764892). Moreover, the Vernalis (R&D) patented V81444 for further trials and commercialization of the drug [118]. Caffeine acts as a selective adenosine A_2A_ receptor antagonist (due to its xanthine property) [119] and reduces neurotoxicity by blocking A_2A_ receptors [120]. In 2014, McGill University Health Centre performed the phase III trial of caffeine for PD treatment (Table 1) (NCT01738178). The trial result shows that caffeine did not provide clinically efficient improvement of motor manifestations in 60 PD patients [57]. Based on the available data, V81444 might be an effective Adenosine A_2A_ antagonist compared to caffeine treatment. However, the epidemiologic links between caffeine and lower PD risk do not appear to be explained by symptomatic effects [57].

### 2.14. Antioxidants and Botanical-Based Medication

The free radical scavenging activity of antioxidants is the major contributor to the protection of dopaminergic neurons and improved mitochondrial functions in sporadic and hereditary PD [121]. Mitochondrial dysfunction is associated with PD pathogenesis; free radical scavenging activity eliminates damaged mitochondria by mitophagy and provides neuroprotection in PD [122]. The decrease of reduced glutathione in the PD brain is one of the pathogenic hallmarks of neuroinflammation [123]. It indicates the importance of reduced glutathione therapy against the oxidation of neurons in the PD treatment. Intranasal application of reduced glutathione (tripeptide glutathione) is a new method of administration used in the antioxidant treatment. We found two clinical trials that use intranasal glutathione as antioxidant therapy for PD treatment in phase I. The status of intranasal glutathione therapy for thirty-four PD subjects shows it is complete without results (NCT01398748). In addition, the trial status of intranasal reduced glutathione therapy for 15 PD subjects also shows it is complete without trial results (NCT02324426). The reduced glutathione treatment modulates the excessive free radical formation and inhibits neuroinflammation in PD [124].

In herbal extract treatment, we observed two clinical trials (Win-1001X and hypoestoxide) for PD treatment. Win-1001X is a plant-based herbal extract for PD treatment in phase II, developing by Medi Help Line. Win-1001X contains the extracts of three plants, for instance, *Angelica tenuissima Nakai*, *Dimocarpus longan* (L.), and *Polygala tenuifolia*. Kim et al. (2014) revealed that Win-1001X increased LC3-II/I, DOR, and GATE16 autophagy-related protein expressions in the midbrain and rescued neuronal damage in rodents [125]. Furthermore, they showed that Win-1001X extract mainly targets autophagy and antioxidant mechanisms and reduced neuroinflammation [125]. This clinical trial used WIN-1001X (400, 800, and 1200 mg) to treat early PD patients (Table 2) (NCT04220762). However, the trial status of WIN-1001X therapy shows subject recruitment with no posted results (NCT04220762). Hypoestoxide is a natural active diterpene phytochemical constituent of *Hypoestes rosea* that acts against PD progression and cancer [126]. Ojo-Amaize and Cottom, (2016) revealed that hypoestoxide offers several beneficial effects in inhibiting the activity of IκB, NFκB, and other inflammatory pathways and modulate PD features [126]. Hypoestoxide is undergoing phase I/II clinical trial for PD by Adesola Ogunniyi, University of Ibadan (Table 2) (NCT04858074). Besides, preclinical data also showed improvements in motor symptoms and lowered disease progression of PD [127].

However, the advantages of herbal extract treatment include no serious adverse events and long-term benefits in disease management compared to modern medicine.

### 2.15. Others

We found two phase-II clinical trials that use other types of drugs for the PD treatment, i.e., GM 608, and NLY01. GM 608 is a motoneuronotrophic endogenous embryonic neural regulatory and signaling peptide (synthetic oligopeptide) developing by Genervon Biopharmaceuticals, LLC. GM 608 has neuroprotection and regulates the development of the human nervous system [128]. The status of the GM 608 phase II trial shows completed in subject recruitment, but no published data is available (Table 1) (NCT01850381). NLY01 is a novel exenatide-based compound developing by Neuraly Inc. [129]. The NLY01 is a pegylated form of exendin-4 (exenatide), which binds to glucagon-like peptide-1 receptors (GLP-1R) and expresses in glial brain cells [129]. Studies of NLY01 in PD showed that NLY01 limited neuronal death decreased formation of an inflammatory cascade and neurotoxic astrocytes, and partial motor function decline [129]. NLY01 is in a phase II trial; the status is under recruitment which is developing by Neuraly, and they estimate to complete it by December 2022 (Table 1) (NCT04154072).

## 3. Discussion

The incidence of PD is increasing with age; the PD patient number may be doubled worldwide by 2040 [130]. Still, there is no permanent cure available for PD patients [122]. Additionally, no clear diagnostic method exists to evaluate the PD [131]. Therefore, it is important to search for the mechanisms or diagnosis/screening of PD. Preclinical data are exceedingly important for every human clinical trial [132]. Preclinical studies involve a wide range of studies (*in vitro* or *in vivo*) for the safety, toxicity, and mechanisms of the PD and provide information on dosage safety and potential toxicity. Several preclinical *in vitro* or *in vivo* investigations effectively reduced or protected from the PD symptoms, but most of them are not yet tested on humans, or some failed when tested in humans [19]. Even though levodopa treatment is a gold standard to improve PD symptoms, levodopa has no neuroprotection [133]. Levodopa therapy will become ineffective after 5 years of usage in PD patients [34]. Combination therapy has emerged as an alternative therapy and has shown greater benefits than monotherapy [134]. In PD, reducing the dose of levodopa and its use combined with other drugs temporarily benefits motor symptoms (for instance, dopamine agonists or monoamine oxidase-B inhibitors). Drugs such as levodopa, carbidopa, monoamine oxidase-B inhibitors, catechol-O-methyl transferase inhibitors, apomorphine were established over 3–5 decades ago [134,135,136]. Drug combinations (carbidopa–levodopa and carbidopa–entacapone–levodopa) are used for PD treatment, but the drug combination is expensive [137]. Duodopa, a micro-suspension gel of levodopa and carbidopa used for continuous stimulation of dopaminergic cells in PD patients, costs over USD 35,000/year/patient [9]. Several non-pharmacologic surgical treatments are also available, such as deep brain stimulation (DBS) and focused ultrasound therapy [138,139,140,141]. DBS treatment is used in patients with severe motor symptoms who are unresponsive to levodopa and carbidopa therapy for a minimum of 4 years [138,139,140,141]. DBS involves implanting electrodes in the patient’s brain to improve PD motor symptoms; DBS risks include brain hemorrhage, device malfunction, infection, and mental illness with side effects such as imbalance, impaired vision, or vocal problems [138,139,140,141]. Moreover, the actual underlying mechanisms of DBS treatment are not yet known [142]. Additionally, DBS is expensive at about USD 50,000/patient, which is not affordable for all PD patients in low or middle-income countries [143].

Although the dopamine receptor agonists play an important role in PD symptomatic treatment, prolonged usage of dopamine receptor agonists causes side effects such as drowsiness, illusions, orthostatic hypotension, compulsive eating, weight gain, and edema [144,145,146]. Besides, studies showed that long-term usage of dopamine receptor agonists in PD patients caused several psychotic behavioral side effects such as gambling behavior and hypersexuality [147,148]. The adverse effects are dose-dependent and may sensitize/damage the dopaminergic receptors in the basal ganglia permanently [149]. In addition, physicians should be aware that compulsive eating resulting in significant weight gain may occur in PD as a side-effect of dopamine agonist medications such as pramipexole [144].

Lewy body dementia is the most common hallmark of neurodegenerative diseases. Chia et al. (2021) performed whole-genome sequencing in controls and Lewy body dementia cases (Alzheimer’s and Parkinson’s diseases) to find out the genetic architecture [150]. Their study revealed that *GBA1*, *BIN1*, *TMEM175*, *SNCA-AS1*, and *APOE* loci are responsible for increases in Lewy body pathogenesis [150]. PD is a multigene risk associated with multiple-genetic changes. Nevertheless, recent studies showed that gene therapy also causes several adverse effects such as undesirable immune system reactions, inflammation, organ failure, and cancer [151].

Health risks of plasma transfusions include HIV, hepatitis C, or hepatitis B viral infections [152,153]. To avoid such risks, donor rested plasma or pathogen reduced plasma are safer [154]. Donor rested plasma is safe due to quarantine (subsequent donations) and tests for infectious diseases [154,155]. In addition, pathogen-reduced plasma is also safe; it increases protection from lipid-enveloped viruses [154,156]. However, plasma therapy also can cause risks associated with non-enveloped viral infections (e.g., hepatitis A, parvovirus B19) and prion diseases [157]. Several IVIG injected cases acquired acute hepatitis in the United States, Europe, Norway, and Puerto Rico [158]. Moreover, stored plasma is also a risk factor in plasma therapy; for instance, few previous cases also found bacterial infections of *Pseudomonas, Staphylococcus*, *Klebsiella*, *Providencia rettgeri,* and *Propionibacterium*, or even it may cause a risk of immune suppression [159].

In the present review, we found 293 PD clinical trials on ClinicalTrials.gov (active as of 16 June 2021). Next, we shortlisted forty-seven clinical trials as novel PD drugs or therapies (excluded levodopa/carbidopa/add-on therapies). Among them, 19 trials are in phase I (41%), 25 clinical trials are in phase II (53%), and 3 trials are in phase III (6%). The PD therapeutic strategy of each clinical trial shows 29, 5, 1, 5, 5, and 2 clinical trials use small molecules, monoclonal antibodies, plasma therapy, cell therapies, gene therapies, and herbal extracts, respectively. The majority of PD clinical trials use small molecular drugs (29 clinical trials, 61.7%), but most of them are monotherapy, not multi-target. All the therapeutic strategies in these clinical trials are divided into 15 types, based on the type of drug/therapy. These 15 types include (1) dopamine receptor agonists: 6 trials, (2) anti-α-synuclein aggregation therapy: 7 trials, (3) convalescent plasma therapy: 1 trial, (4) cell-based therapy: 5 trial, (5) gene therapy: 6 (5 gene therapy and 1 small molecular glucosylceramide synthase inhibitor) (6) serotonin receptor agonists or antagonists: 3 trials, (7) muscarinic and nicotinic acetylcholine receptor agonists: 3 trials, (8) monoamine reuptake inhibitors: 1 trial, (9) NMDAR modulators: 2 trials, (10) anti-apoptotic drugs: 2 trials, (11) kinase inhibitors: 2 trials, (12) myeloperoxidase inhibitors: 1 trial, (13) adenosine A_2A_ receptor antagonists: 2 trials, (14) antioxidants/botanical-based medication: 4 trials, and (15) others: 2 trials (Figure 5).

Drug discovery is an expensive, slow, and risky business. The overall failure rate in drug development is more than 96%, in which 90% fail during clinical development [160]. From the forty-seven clinical trials in the present study, 44 trials (94%) are in phase I for safety and dosage evaluations or phase II for efficacy and side effect assessments. Only three trials (6%) in phase III are active (embryonic cell implant surgery, sarizotan, and caffeine) for efficacy and adverse effect monitoring. It indicates the need for more drugs in clinical trials for PD treatment. In addition, the available medication or treatment is not affordable for low/middle-income people. The cost of PD medications alone is around $5853.50 USD/patient/year in Brazil, and DBS is around $186,244 USD/patient/5-years [161].

According to Parkinson’s Foundation, about 10-million people worldwide live with PD, and PD incidence increases with age [162]. The disease progression and symptoms of PD are unique from one patient to another, and their medications are decided based on the disease progression and symptoms of each patient. Overall, the review highlights the current treatments for PD in clinical trials updated as of 16 June 2021. However, the low success rate of curable PD drugs, and high PD incidence, alarming the need to discover multi-target or combined (disease-modification/neuroprotective) medications for the PD.

## 4. Conclusions

In conclusion, the treatments that use small molecule α-synuclein aggregation therapy (ambroxol or Cu(II)ATSM) or monoclonal antibody (AFFITOPE^®^ PD01A, and PRX002) or gene therapy (PR001A/AAV-GAD) might be promising compared to other clinical trials/therapies for the treatment of PD in future. In our view, the aim of the clinical trials should delay motor complications in later stages that may show long-lasting complications. However, with the rapid rise of the rate of PD incidence worldwide, finding new multi-target drugs or therapies without adverse reactions is a huge challenge.

## Figures and Tables

**Table 1:** Small molecule drugs in phase I, II, or III clinical trials for PD treatment. The data are based on the trial status (ongoing, updated, or discontinued) on ClinicalTrials.gov (https://clinicaltrials.gov) that is active as of 16 June 2021.

Therapeutic Strategy	Classification	Name	PD Subjects	Trial Status	Reasons for Discontinuation	Sponsor	ClinicalTrials.gov Identifier and Reference
Dopamine receptor agonists	Small molecular Dopamine D_1_/D_5_ partial agonist	PF-06412562	Advanced-stage PD	Phase I	Not applicable	Milton S. Hershey Medical Center	NCT03665454 [30]
Dopamine receptor agonists	Small molecular Dopamine D_1_ partial agonist	PF-06669571	Idiopathic PD	Phase I	Not applicable	Pfizer	NCT02565628 [31]
Dopamine receptor agonists	Small molecular Dopamine D_1_/D_5_ partial agonist	PF-06649751	Idiopathic PD	Phase I	Not applicable	Pfizer	NCT02224664 [32]
Dopamine receptor agonists	Small molecular Dopamine D_1_/D_5_ partial agonist	PF-06649751	Early stage PD	Phase II discontinued	Terminated due to lack of efficacy in moderate/advanced PD.	Pfizer	NCT02847650 [33]
Dopamine receptor agonists	Small molecular Dopamine D_1_/D_5_ partial agonist	PF-06649751	PD with motor fluctuations	Phase II discontinued	Terminated due to insufficient efficacy.	Pfizer	NCT02687542
Dopamine receptor agonists	Small molecular Dopamine D_2_ agonist	CJH1 (CLR4001)	PD patients	Phase I/II	Unknown	Alexandra Marine and General Hospital	NCT01684475
Anti-α-synuclein aggregation therapy	Small molecular Dock with β-glucocerebrosidase to increase its levels and decrease the cerebrospinal fluid α-synuclein level	Ambroxol	PD with dementia	Phase II	Not applicable	Lawson Health Research Institute	NCT02914366 [49]
Anti-α-synuclein aggregation therapy	Small molecular Peroxynitrite scavenger	Cu(II)ATSM	Early idiopathic PD	Phase I	Not applicable	Collaborative Medicinal Development Pty Limited	NCT03204929
Gene therapy	Small molecular glucocerebrosidase (*GBA*) gene mutating therapy	GZ/SAR402671	Early stage PD	Phase II discontinued	Terminated due to not meeting the primary and secondary endpoints.	Genzyme	NCT02906020 [53]
Serotonin receptor agonists or antagonists	Small molecular Dual 5-HT_6/5_-HT_2_ antagonist	SYN120	PD with dementia	Phase II	Not applicable	Biotie Therapies Inc.	NCT02258152
Serotonin receptor agonists or antagonists	Small molecular Selective 5-HT_1A_ partial agonist	Piclozotan (SUN N4057)	Idiopathic PD	Phase II	Not applicable	Daiichi Sankyo, Inc.	NCT00623363
Serotonin receptor agonists or antagonists	Small molecular Selective 5-HT_1A_ agonist and D_2_ antagonist	Sarizotan	Idiopathic PD	Phase III	Not applicable	EMD Serono	NCT00105508 [54]
Monoamine reuptake inhibitors	Small molecular Triple monoamine reuptake inhibitor (serotonin, noradrenaline, and dopamine reuptake inhibitor)	NS 2330 (tesofensine)	Early stage PD	Phase II	Not applicable	Boehringer Ingelheim	NCT00148486
Muscarinic and nicotinic acetylcholine receptor agonists	Small molecular Muscarinic agonist and sigma1 agonist	ANAVEX2-73	PD with dementia	Phase II	Not applicable	Anavex Life Sciences Corp.	NCT04575259
Muscarinic and nicotinic acetylcholine receptor agonists	Small molecular Nicotinic agonist	Nicotine transdermal patch	Early stage PD	Phase II	Unknown	James BOYD MD	NCT01560754
Muscarinic and nicotinic acetylcholine receptor agonists	Small molecular Nicotinic agonist	Nicotine nasal spray	PD (Hoehn and Yahr stage 2–3)	Phase II	Not applicable	El Instituto Nacional de Neurologia y Neurocirugia Manuel Velasco Suarez	NCT03865121
N-methyl-D-aspartate receptor (NMDAR) modulators	Small molecular NMDAR modulator	NYX-458	Mild cognitive impairment associated with PD	Phase II	Not applicable	Aptinyx	NCT04148391
NMDAR modulator	Small molecular D-amino acid oxidase inhibitor	DAAOI-P	PD with dementia	Phase II	Not applicable	China Medical University Hospital	NCT04470037
Anti-apoptotic drugs	Small molecular Dibenz[b,f]oxepin-10-ylmethyl-prop-2-ynyl-amine, hydrogen maleate salt	TCH346	Early stage PD	Phase I/II	Not applicable	Novartis	NCT00407212
Anti-apoptotic drugs	Small molecular Synthetic tetracycline derivative	Minocycline	Early stage untreated PD	Phase II	Not applicable	University of Rochester	NCT00063193 [55]
Kinase inhibitors	Small molecular Semisynthetic inhibitor of the mixed lineage kinase family	CEP-1347 (KT7515)	Early stage PD	Phase II/III discontinued	Terminated due to insufficient efficacy.	Cephalon	NCT00040404 [56]
Kinase inhibitors	Small molecular Orally selective inhibitor of cABL protein tyrosine kinase	K0706	Early stage PD	Phase II	Not applicable	Sun Pharma Advanced Research Company Limited	NCT03655236
Myeloperoxidase inhibitors	Small molecular Irreversible myeloperoxidase inhibitor	AZD3241	Idiopathic PD	Phase II	Not applicable	AstraZeneca	NCT01603069
Adenosine A_2A_ receptor antagonists	Small molecular Adenosine A_2A_ antagonist	V81444	PD patients	Phase I	Not applicable	Vernalis (R&D) Ltd.	NCT02764892
Adenosine A_2A_ receptor antagonists	Small molecular Selective Adenosine A_2A_ antagonist	Caffeine	PD (Hoehn and Yahr stage 1–3)	Phase III	Not applicable	McGill University Health Centre/Research Institute of the McGill University Health Centre	NCT01738178 [57]
Antioxidants	Small molecular Intranasal glutathione therapy	Reduced glutathione	PD (modified Hoehn and Yahr stage < 3)	Phase I	Not applicable	Bastyr University	NCT01398748
Antioxidants	Small molecular Intranasal reduced glutathione	Reduced glutathione	PD (Hoehn and Yahr stage 2–3)	Phase I	Not applicable	University of Washington	NCT02324426
Others	Small molecular Synthetic oligopeptide	GM 608	Mild to moderate-stage PD	Phase II	Not applicable	Genervon Biopharmaceuticals, LLC	NCT01850381
Others	Small molecular Glucagon-like peptide 1 receptor agonist	NLY01	Early stage PD	Phase II	Not applicable	Neuraly, Inc.	NCT04154072

**Table 2:** Monoclonal antibodies and vaccines, plasma therapy, cell therapy, gene therapy, and herbal extracts in phase I, II, or III clinical trials for PD treatment. The data are based on the trial status (ongoing, updated, or discontinued) on ClinicalTrials.gov (https://clinicaltrials.gov) that is active as of 16 June 2021.

Therapeutic Strategy	Classification	Name	PD Subjects	Trial Status	Reasons for Discontinuation	Sponsor	ClinicalTrials.gov Identifier and Reference
Anti-α-synuclein aggregation therapy	Monoclonal antibody	ABBV-0805	Idiopathic PD	Phase I discontinued	Withdrawn due to strategic considerations.	AbbVie	NCT04127695
Anti-α-synuclein aggregation therapy	Vaccine Short synthetic peptides	AFFITOPE^®^ PD01A	Early stage PD	Phase I	Not applicable	Affiris AG	NCT01568099 [45]
Anti-α-synuclein aggregation therapy	Monoclonal antibody IgG_1_ protein produced from memory B cells	BIIB054	PD patients	Phase II discontinued	Terminated due to lack of efficacy.	Biogen	NCT03318523
Anti-α-synuclein aggregation therapy	Monoclonal antibody	PRX002 (Prasinezumab/ RO7046015)	Idiopathic PD	Phase I	Not applicable	Prothena Biosciences Limited	NCT02157714 [43]
Anti-α-synuclein aggregation therapy	Monoclonal antibody	PRX002 (Prasinezumab/ RO7046015)	Early stage PD	Phase II	Not applicable	Hoffmann-La Roche	NCT03100149 [43]
Convalescent plasma therapy	Young plasma infusions	Infusions of young plasma	Moderate-stage PD	Phase I	Not applicable	Stanford University	NCT02968433 [58]
Cell-based therapy	Injection cultured human retinal pigment epithelial cells into both hemispheres	Spheramine/ BAY86-5280	Advanced-stage PD	Phase II discontinued	Terminated. The trial was completed, and only the lifelong extended follow-up phase was discontinued after 12 years.	Bayer	NCT00206687 [62]
Cell-based therapy	Xenotransplantation of immunoprotected (alginate-encapsulated) choroid plexus cells in the brain	NTCELL	Idiopathic PD	Phase I/II	Not applicable	Living Cell Technologies	NCT01734733
Cell-based therapy	Xenotransplantation of immunoprotected (alginate-encapsulated) choroid plexus cells in the brain	NTCELL	Idiopathic PD	Phase II	Not applicable	Living Cell Technologies	NCT02683629
Cell-based therapy	Neuronal progenitor stem cells	Adult neuronal progenitor stem cell	PD	Phase II discontinued	The study was withdrawn before participants were enrolled.	Rajavithi Hospital	NCT00927108
Cell-based therapy	Embryonic dopamine cell implant	Embryonic dopamine cell implant surgery	Idiopathic PD	Phase III	Not applicable	University of Colorado, Denver	NCT00038116 [73]
Gene therapy	AAV2-GDNF delivered to the putamen	AAV2-GDNF	Mild to moderate and moderate to severe PD	Phase I	Not applicable	Brain Neurotherapy Bio, Inc.	NCT04167540
Gene therapy	Surgical infusion of AAV-GAD into the subthalamic nucleus	Glutamic acid decarboxylase (*GAD*) gene therapy	Advanced-stage PD	Phase I	Not applicable	Neurologix, Inc.	NCT00195143 [80]
Gene therapy	Adeno-associated virus delivery of neurturin gene in the substantia nigra and putamen	CERE-120	Idiopathic PD	Phase I/II	Not applicable	Sangamo Therapeutics	NCT00985517 [81]
Gene therapy	Glucocerebrosidase gene therapy by intra cisterna magna administration	PR001A	Moderate to severe PD	Phase I/IIa	Not applicable	Prevail Therapeutics	NCT04127578
Gene therapy	AAV2-neurturin gene therapy	CERE-120	Idiopathic PD	Phase II	Not applicable	Sangamo Therapeutics (Ceregene)	NCT00400634 [83]
Antioxidants and botanical-based medication	Plant-based herbal dry powder	Hypoestoxide	PD	Phase I/II	Not applicable	Adesola Ogunniyi, University of Ibadan	NCT04858074
Antioxidants and botanical-based medication	Plant-based herbal extract	WIN-1001X	Early stage PD	Phase II	Not applicable	Medi Help Line	NCT04220762

## Data Availability

Not applicable.
